# Chemical Reporters and Their Bioorthogonal Reactions for Labeling Protein *O*-GlcNAcylation

**DOI:** 10.3390/molecules23102411

**Published:** 2018-09-20

**Authors:** Eun Ju Kim

**Affiliations:** Department of Science Education-Chemistry Major, Daegu University, Gyeongsan-si 712-714, Gyeongsangbuk-do, Korea; eunkim@daegu.ac.kr; Tel.: +82-10-7127-4089

**Keywords:** metabolic chemical reporters, *O*-GlcNAc, bioorthogonal reactions

## Abstract

Protein *O*-GlcNAcylation is a non-canonical glycosylation of nuclear, mitochondrial, and cytoplasmic proteins with the attachment of a single *O*-linked β-*N*-acetyl-glucosamine (*O*-GlcNAc) moiety. Advances in labeling and identifying *O*-GlcNAcylated proteins have helped improve the understanding of *O*-GlcNAcylation at levels that range from basic molecular biology to cell signaling and gene regulation to physiology and disease. This review describes these advances in chemistry involving chemical reporters and their bioorthogonal reactions utilized for detection and construction of *O*-GlcNAc proteomes in a molecular mechanistic view. This detailed view will help better understand the principles of the chemistries utilized for biology discovery and promote continued efforts in developing new molecular tools and new strategies to further explore protein *O*-GlcNAcylation.

## 1. Introduction

Protein *O*-GlcNAc-modification (*O*-GlcNAcylation) is a post-translational modification (PTM) that involves the attachment of *O*-linked β-D-*N*-acetylglucosamine onto serine (Ser) or threonine (Thr) residues of myriad intracellular proteins [1]. *O*-GlcNAcylation is a one-sugar modification with no elongation into more complex structures that is found in the nucleus, cytoplasm, and mitochondria. These features of *O*-GlcNAcylation make it different from other types of protein glycosylation (i.e., protein *N*- or *O*-glycosylation), which exist mainly as oligosaccharides or polysaccharides in the cell surface and extracellular compartments. *O*-GlcNAcylation is a dynamic process with a wide turnover rate (0.02 h^−1^ to 1.6 h^−1^) [2] and the dynamics of the modification are catalyzed by a single pair of enzymes in a nutrient- and stress-responsive manner. *O*-GlcNAc is added to the whole range of protein substrates by *O*-GlcNAc transferase (OGT) [3] and removed by *O*-GlcNAcase (OGA) [4]. This intracellular form of protein glycosylation affects almost every cellular process ranging from transcription and translation to signal transduction and metabolism [5,6]. A large body of evidence has implicated an alteration of *O*-GlcNAc homeostasis in the pathogenesis of different kinds of chronic diseases; an increase in *O*-GlcNAc levels has been observed in a number of insulin signaling proteins and mitochondrial proteins in diabetic cells [7], as well as in various types of cancer [8]. In contrast, decreased *O*-GlcNAc levels with inversely increased phosphorylation have been observed in the microtubule-associated tau protein, which is believed to promote its oligomerization and lead to Alzheimer’s disease (AD) [9]. Therefore, the characterization of *O*-GlcNAcylated proteins and the dynamics of this modification are essential for a better understanding of the functions of *O*-GlcNAc in human physiology and diseases. On the other hand, the substoichiometric and labile nature of *O*-GlcNAcylation makes its detection and identification quite difficult and even more challenging when the *O*-GlcNAcylated proteins are in low cellular abundance. To tackle these challenges, several innovative strategies and novel chemical reporters in association with their corresponding chemoselective reactions have been developed over the past few years. This review discusses the recent progress in labeling methods of protein *O*-GlcNAcylation, including synthetic chemical reporters and their chemoselective reactions, in a mechanistic view.

## 2. Metabolic Chemical Reporters and Their Chemistries for Labeling *O*-GlcNAc Proteins

**Figure 1:**
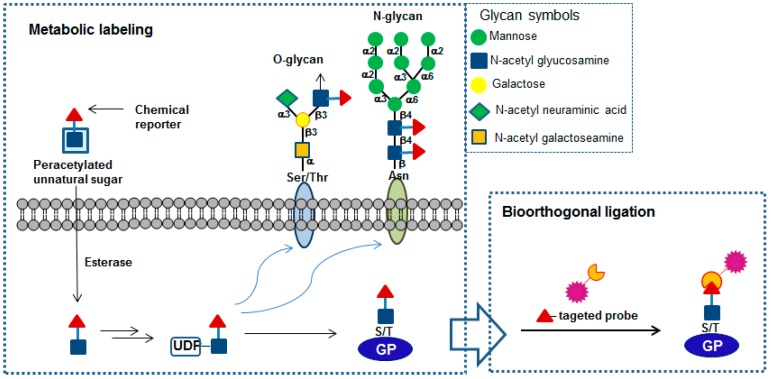
Schematic description of detection and isolation using metabolic labeling followed by bioorthogonal ligation. GP represents a glycoprotein.

Metabolic chemical reporters (MCRs) are unnatural chemical functionalities with unique reactivity and they can be introduced into naturally occurring biomolecules of a living system, generally through the cell’s biosynthetic machinery. These chemical reporters are then reacted with specifically designed molecular probes in bioorthogonal labeling reactions that allow the visualization and/or isolation of biomolecules of interest. For the successful application of these techniques, chemical reporter groups should be stable before the reaction occurs and nontoxic to living systems. Moreover, the reaction between the chemical reporter groups and the probes must occur selectively under physiological conditions with either of them being inert to the plethora of chemical functionality found in cells (Figure 1). Since Bertozzi et al. originally utilized the versatility of the hexosamine salvage pathway to deliver an azide moiety into *O*-GlcNAcylated proteins [10], other chemical reporters including alkyne [11], alkene [12], and diazirine [13] functionalities have been introduced for *O*-GlcNAc studies. This section describes unnatural sugars containing these chemical reporters for labeling *O*-GlcNAc proteins and their bioorthogonal chemical reactions developed to date. In addition, a brief discussion on how these molecular tools can be used to decipher *O*-GlcNAc functional roles is provided.

### 2.1. Azide- or Terminal Alkyne-Containing Metabolic Chemical Reporters

Azide functionality is the most widely used bioorthogonal group because it is essentially absent from biological systems and its reactivity is orthogonal to the majority of biological functionalities. Azide-bearing unnatural monosaccharides have been incorporated into proteins and used in a range of biological research areas. To date, five types of per-*O*-acetylated GlcNAc and GalNAc analogs containing an azide moiety have been developed and used to label *O*-GlcNAc-modified proteins with an azide functional group: (1) per-*O*-acetylated *N*-azidoacetylglucosamine (Ac_4_GlcNAz) [10]; (2) per-*O*-acetylated *N*-azidoacetylgalactosamine (Ac_4_GalNAz) [14]; (3) per-*O*-acetylated 6-azido-6-deoxy-*N*-acetylglucosamine (Ac_3_6AzGlcNAc) [15]; (4) per-*O*-acetylated 4-deoxy-*N*-azidoacetyl-glucosamine (Ac_3_4dGlcNAz) [16]; and (5) per-*O*-acetylated-*N*-pentynylglucosamine (Ac_4_GlcNAlk) [11].

**Figure 2:**
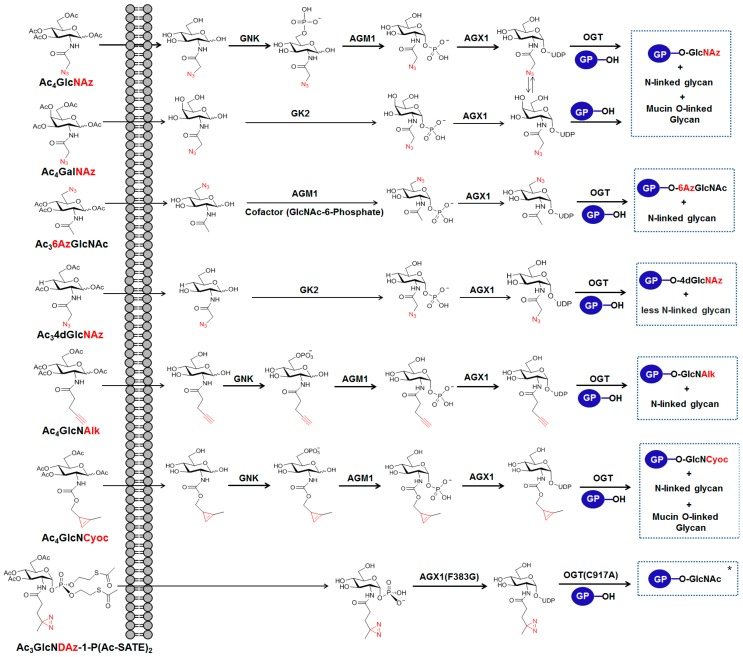
Metabolic chemical reporters (MCRs) of GlcNAc analogs bearing azide, alkyne or cycloproene functionalities and their possible salvage pathways.

Ac_4_GlcNAz is the first azidosugar that has been shown to be converted metabolically to the nucleotide sugar uridine diphosphatidyl GlcNAc analog UDP-GlcNAz. Then, UDP-GlcNAz serves as substrates for OGT allowing its incorporation into glycoprotein substrates (Figure 2 [10]). Zhao et al. used metabolic labeling with Ac_4_GlcNAz and the Staudinger ligation reaction for a global *O*-GlcNAc proteomic study and identified 199 putative *O*-GlcNAcylated proteins with a wide range of functions, suggesting that *O*-GlcNAc is involved in regulating multiple cellular pathways [17]. In addition to the Staudinger ligation, copper-catalyzed alkyne−azide cycloaddition (CuAAC) reaction between an alkyne-functionalized agarose resin and whole-cell lysates from Ac_4_GlcNAz-fed HEK293 cells was also used to identify approximately 1500 putative *O*-GlcNAcylated proteins as well as 185 GlcNAcylation sites [18]. Natural UDP-GlcNAc is used for GlcNAc incorporation not only into *O*-GlcNAc proteins, but also into the *N*-linked glycans and mucin-type *O*-linked oligosaccharides. In addition, the interconversion of UDP-GlcNAc to UDP-GalNAc can lead to the incorporation of GlcNAc at the core of mucin-type *O*-linked glycans. Similarly, GlcNAz is incorporated metabolically into *O*-GlcNAc proteins, but also *N*-linked and/or mucin-type *O*-linked glycans are labeled.

Ac_4_GalNAz, which was originally developed for metabolically labeling mucin-type *O*-linked glycans [19], has also been used in labeling *O*-GlcNAc proteins because UDP-GalNAz is found to be epimerized efficiently to give UDP-GlcNAz by the enzyme UDP-galactose 4-epimerase (GALE) [14]. An application of metabolic labeling with Ac_4_GalNAz and the Staudinger ligation showed that *O*-GlcNAcylation could be a cotranslational process and provides a mechanism for protecting nascent polypeptide chains from premature degradation by decreasing ubiquitination [20].

Pratt et al. examined metabolic labeling with Ac_4_GlcNAlk, the terminal alkynyl analog of GlcNAc and showed that GlcNAlk can be incorporated into *O*-GlcNAc proteins under low-glucose growth conditions [11]. In their studies, GlcNAlk did not appear to be interconverted to GalNAlk, thereby avoiding mucin *O*-linked glycoproteins’ labeling even though it labels *N*-linked glycans [11]. Gurel et al. applied GlcNAlk metabolic labeling and the CuAAC reaction method to examine the role of *O*-GlcNAcylation in diabetic retinopathy [21]. A previous study shows that a high glucose-mediated increase in *O*-GlcNAcylation in retinal pericytes impaired their migration, which plays an important role in constructing healthy capillaries in the retina [22]. The authors discovered that the retinal pericytes responded with a large increase in *O*-GlcNAcylation compared to other retinal vascular cells under the conditions of either high glucose or the presence of an OGA inhibitor, resulting in elevated apoptosis of retinal pericytes [21]. In addition, they identified approximately 34 *O*-GlcNAcylated proteins that are involved in the cell death processes and found that the increased *O*-GlcNAcylation of p53 was associated with its increased protein level in retinal pericytes, supporting the role of elevated *O*-GlcNAcylation in the selective loss of retinal pericytes during diabetes [21].

The GlcNAc analogs described above have a limitation of “off-target” labeling necessitating careful data analysis. To address this limitation, Pratt et al. explored 6AzGlcNAc and showed the exclusive labeling of intracellular proteins with a background labeling level of mucin *O*-linked glycoprotein [15]. 6AzGlcNAc bypasses the first step of phosphorylation at the 6-hydroxyl of the sugar by *N*-acetylglucosamine kinase (GNK) and directs the phosphorylation of its 1-hydroxyl by the phosphoacetylglucosamine mutase (AGM1) enzyme in the presence of a cofactor, such as GlcNAc-6-phosphatein in the process of its conversion to UDP-6AzGlcNAc [15]. An *O*-GlcNAc proteomic study of NIH3T3 cells using this MCR and CuAAC reaction method led to the identification of 366 6AzGlcNAc-labeled proteins, 350 of which were annotated as intracellular proteins [15]. Although the probe, Ac_3_6AzGlcNAc, also labels *N*-linked glycans, it exhibits improved selectivity in labeling *O*-GlcNAc proteins. On the other hand, the labeling efficiency with Ac_3_6AzGlcNAc is low because it cannot be processed by canonical GlcNAc salvage pathway.

Recently, a new GlcNAc analog, Ac_3_4dGlcNAz, lacking the hydroxyl at C4 was reported [16]. The probe, 4dGlcNAz, has been proposed to be processed by the GalNAc salvage pathway involving GalNAc kinase 2 (GK2) and AGX1 to form UDP-4dGlcNAz [16]. The majority of glycosidic linkages to GlcNAc or GalNAc in glycoconjugates, such as *N*-linked/*O*-linked glycans, link to the C4 hydroxyl group of the sugar. Therefore, the lack of a hydroxyl at C4 of this probe greatly reduces its incorporation into the cell surface glycoconjugates. In addition, a previous study reported that 4dGalNAc is not a substrate of the polypeptide α-GalNAc transferase T1 (ppGalNAc-T1) to produce *O*-linked glycan proteins [23]. These features allow 4dGlcNAz to have a higher selectivity over previous metabolic chemical reporters for *O*-GlcNAcylation. Metabolic labeling of proteins can be removed by endogenous OGA, which attenuates the labeling efficiency. In particular, *O*-4dGlcNAz modification was reported to increase the resistance to OGA hydrolysis, leading to enhanced labeling efficiency for *O*-GlcNAcylation [16]. Proteome wide analysis of *O*-GlcNAcylated proteins in HEK293 cells using this OGA-resistant MCR and CuAAC reaction discovered 507 putative *O*-GlcNAcylated proteins including 281 previously known and 266 novel ones, 484 of which were annotated as intracellular proteins [16].

### 2.2. Chemoselective Reactions Involving Azide-Functionality

**Figure 3:**
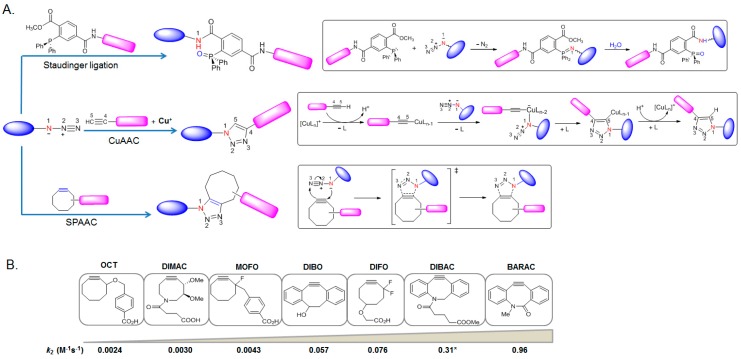
Bioorthogonal reactions with azide (**A**); and a series of cycoalkyne reagents developed for SPAAC with corresponding *k*_2_ values for the 1,3-dipolar cycloaddition with benzyl azide (**B**). Simplified mechanistic views of each reaction (Staudinger ligation in the upper, CuAAC in the middle, SPAAC in the bottom in (**A**)) are described in the box.

Figure 3 shows the most widely used orthogonal chemistries with azide or alkyne functionality. The Staudinger ligation and CuAAC reactions have been reviewed thoroughly previously [24]; this section briefly discusses these reactions.

Staudinger ligation: Azide-bearing proteins have been reacted with arylphosphines to finally form a stable amide bond in Staudinger ligations (Figure 3A, upper) [25]. This reaction has been applied to label biomolecules in a variety of biological systems [26,27] and its mechanistic details have been reviewed thoroughly [24]. The Staudinger ligation, however, has slow reaction kinetics with second-order constants in the range of 10^−3^ M^−1^ s^−1^ [28]. In addition, phosphine reagents tend to oxidize in the presence of air or metabolic enzymes, thereby requiring a high concentration of reagents [29].

Copper-catalyzed alkyne-azide cycloaddition (CuAAC): Azide also reacts with terminal alkynes in the presence of a Cu(I) catalyst to form a conjugation product of 1,2,3-triazole (Figure 3A, middle) [30]. The CuAAC reaction is a regioselective conjugation reaction, giving only 1,4-regioisomer product of 1,2,3-triazole and is second order with respect to copper [31]. The rates depend on the amount of Cu(I) and its coordinating ligands [32] used but the reaction generally proceeds considerably faster (*k*_2_ = 10~100 M^−1^ s^−1^) in the physiological conditions [33]. Owing to its fast and effective bioconjugation of an azide-labeled biomolecule with an appropriate tag, CuAAC has been used widely in numerous studies including proteomic studies [10,18]. On the other hand, the toxicity and undesirable perturbation in the cellular function that copper complexes introduce limits its broad applicability in a living system [34].

Strain-promoted alkyne-azide cycloaddition (SPAAC): SPAAC was developed to eliminate the use of a cytotoxic Cu(I) catalyst. In SPAAC, the rate of alkyne-azide cycloaddition is accelerated with respect to the reaction without Cu(I) by introducing ring strain into the alkyne [35]. The first example of SPAAC is a cyclooctyne (called OCT) and the reaction proceeds as a standard concerted 1,3-dipolar cycloaddition to produce a regioisomeric mixture of triazoles (Figure 3A, bottom) [33,35]. OCT reacts with azide under physiological conditions without toxicity but its kinetics (rate constant (*k*_2_) of 0.0024 M^−1^ s^−1^) are similar to the Staudinger ligation [33,35]. Further optimization has led to the development of a series of structurally varied cyclooctyne-based probes that display differential reactivities through either stain or electronic modulation. The electronic modulation of cyclooctyne scaffolds utilizes electron-withdrawing fluorine atom(s), which has an effect on lowering the LUMO energy of the alkyne and the HOMO–LUMO gap, thereby increasing the reaction rate [33,36]. Strain-modulated cyclooctyne derivatives have two aryl rings fused to a cylcooctyne structure, enhancing the ring strain, which increases the reaction rate. Some examples are dibenzocyclooctyne (DIBO) [37], dibenzoazacyclooctyne (DIBAC) [38], and biarylazacyclooctyne (BARAC) [39]. The cyclooctyne system has been tuned further by employing a heteroatom or a *sp*^2^-like center, such as an amide bond to the cyclooctyne ring structure to improve the water solubility or to impart additional strain to the ring, as shown in the examples of dimethoxycyclooctyne (DIMAC) [40] and BARAC [39]. The optimization of bioorthogonal reagents requires a delicate balance between the reactivity, chemical stability, and selectivity to minimize their off-target [41] reactivity; Figure 3B describes several cycloalkyne derivatives used in biomedical research areas. The SPAAC reaction rates with these reagents lies in the middle of Staudinger ligation and CuAAC reaction (*k*_2_ = 10^−2^ to 1 M^−1^ s^−1^).

### 2.3. Cycloalkene-Containing Metabolic Chemical Reporter

**Figure 4:**
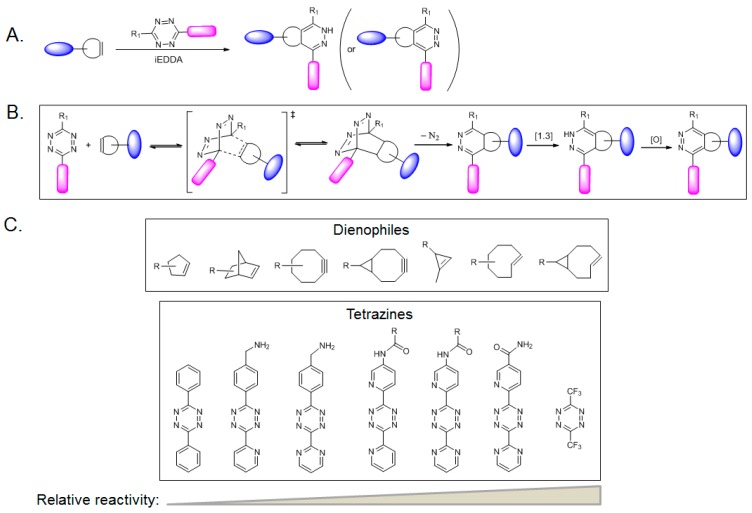
Tetrazine ligation of alkene-functionalized biomolecules through iEDDA: Schematic representation of: iEDDA (**A**); its mechanism (**B**); and several dienophiles and tetrazine derivatives with their relative reactivities (**C**).

Inverse-electron demand Diels–Alder (iEDDA) reactions between diene 1,2,4,5-tetrazines and dienophile alkene or alkyne dienophiles have emerged as bioorthogonal, and metal-free “click” chemistry (Figure 4A) [42]. Since iEDDA was applied to metabolic chemical reporter engineering, several dienophiles, such as alkenes [43], isonitriles [44], and cyclopropenes [45], have been incorporated in carbohydrate derivatives. Owing to the small size and high reactivity of the cyclopropenyl group, the iEDDA reaction between cyclopropene tags and tetrazines has become a popular ligation reaction for detecting and isolating the biomolecules of interest [46,47]. One such study employs the cyclopropene GlcNAc analog Ac_4_GlcNCyoc as a metabolic chemical reporter for *O*-GlcNAc protein labeling (Figure 2) [12]. Ac_4_GlcNCyoc has been shown to label intracellular *O*-GlcNAc proteins as well as the lesser labeling of extracellular glycans [12]. An important aspect of the iEDDA reaction is that it can be orthogonal to azide-alkyne cycloaddition. Therefore, a cycloproene-containing chemical reporter could be combined with an azide-containing chemical reporter to achieve dual labeling of different targets in cells within a single experiment. Continued improvement in the specificity and incorporation efficiency of metabolic probes will advance their applications in *O*-GlcNAc biology.

### 2.4. Chemoselective Ligation Reactions Involving Strained Alkene (or Alkyne) with Tetrazine through iEDDA Reaction

Tetrazine is composed of a six-membered aromatic ring with four nitrogen atoms and its ligation chemistry was first reported in 2008 [48,49]. Among several tetrazine isomers, 1,2,4,5-tetrazine is used most often in tetrazine ligation. A reaction of 1,2,4,5-tetrazine with strained alkenes or alkynes uses an “inverse” electron-demand Diels–Alder reaction, in which dienes (tetrazines) are electron-deficient due to electron withdrawing substituents (dienes; Ψ_3_ is considered the lowest unoccupied molecular orbital), whereas dienophiles (alkenes or alkynes) are electron-rich due to electron donating substituents (dienophiles; Ψ_2_ is considered the highest occupied molecular orbital). Figure 4B represents the mechanism of the iEDDA-initiated conjugation [42]. This begins with a Diels–Alder [4+2] cycloaddition to produce a highly strained cyclic adduct and the adduct then proceeds rapidly via a retro-Diels–Alder reaction to release nitrogen gas and form the corresponding 4,5-dihydropyridazine, which is then converted to the corresponding 1,4-dihydro -isomer through 1,3-prototropic isomerization. Stable 1,4-dihydropyridazine requires an oxidant to be converted to the pyridazine. In contrast, when the alkyne is used as a dienophile, the pyridazine is formed without the additional oxidants. In the iEDDA reaction, the rates depend on the nature of the alkenes and the nitrogen contents of substituents on the tetrazine scaffold and their relative reactivities are shown in Figure 4C.

The iEDDA rates are extraordinarily high, reaching up to 10^5^ M^−1^ s^−1^ when trans-bicyclo[6.1.0]nonene is used as the dienophile [50], which is more than five orders of magnitude higher than SPAAC. In addition, fluorescence-quenching mechanism can be exploited in the tetrazine ligation through iEDDA. In this approach, tetrazines are conjugated to some fluorophores, where fluorescence is quenched by tetrazine, but quenched fluorescence is released to give its fluorescence upon tetrazine ligation through iEDDA [51,52]. Tetrazines generally have high reactivity, particularly those with a high nitrogen content. Therefore, their synthesis is advised to be performed in a well-ventilated hood. The prices and availability of the precursors of tetrazines have resulted in their synthesis on a small scale. On the other hand, their fast reaction rates, bioorthogonality, and mutual orthogonality with other click reactions will allow this chemistry to be used widely in a variety of biological research including protein *O*-GlcNAcylation.

### 2.5. Diazirine-Containing Metabolic Chemical Reporter

Diazirines are a class of cyclopropene ring with the replacement of two double-bonded carbons of the ring with two nitrogen atoms. Diazirines form reactive carbenes upon irradiation with ultraviolet light (UV), which can be inserted into C-H, N-H, or O-H bonds. The diazirine functionalities are generally used to crosslink the neighboring molecules covalently in the construction of an interaction network between a protein of interest and its binding partners. The elucidation of interacting proteins is an important aspect of understanding of protein functions. However, glycan binding proteins are difficult to isolate because of their low binding affinities and the short duration of the complex they form [53]. One approach to tackle this obstacle is to stabilize glycoprotein interactions with the corresponding binding partners by forming a covalent bond between them using photoactivatable cross-linking reagents. On the other hand, photoactivatable cross-linking functionality can be introduced to a monosaccharide, which is then incorporated metabolically into the proteins of interest to identify their interacting proteins. Kohler et al. used diazirine as a photoactivatable cross-linking functional group and introduced this functional group to GlcNAc to identify the binding partners of *O*-GlcNAcylated proteins [13]. Despite this, the first diazirine derivative of GlcNAc, per-*O*-acetylated *N*-diazirine-acetylglucosamine (Ac_4_GlcNDAz), was not a compatible substrate for the GlcNAc salvage pathway [13]. To address this issue, they introduced a precursor of diazirine-carrying GlcNAc-1-phosphate, which was referred to as Ac_3_GlcNDAz-1-P(Ac-SATE)_2_, where 1-*O*-phosphate is protected with two *S*-acetyl-2-thioethyl (Ac-SATE) groups to produce its corresponding UDP-GlcNAc analog carrying diazirine (Figure 2). In addition, the authors mutated the enzyme, AGX1, which converts GlcNAc-1-phosphate to UDP-GlcNAc to enhance the conversion efficiency of the azirine-containing analog [13]. They further optimized by mutating the natural OGT, which originally favors UDP-GlcNAc over UDP-GlcNDAz to its mutant OGT(C917A), which prefers UDP-GlcNDAz over its natural nucleotide sugar, leading to significantly improved *O*-GlcNDAz incorporation [54]. The diazirine-bearing GlcNAc analog is resistant to hydrolysis by OGA, resulting in the accumulation of *O*-GlcNDAz-modified proteins in cells. The ability to efficiently capture interacting proteins by forming covalent bonds with *O*-GlcNAc will offer a useful tool to globally profile glycan binding proteins for the functional characterization of *O*-GlcNAcylation. For instance, nucleoporins that contain phenylalanine and glycine peptide repeats are heavily *O*-GlcNAcylated, but the function of this modification is unclear. Through the use of a diazirine-containing GlcNAc analog, Yu et al. provided evidence on a direct interaction between the nucleus transporter factor-1 and nucleoporins NUP153 and NUP358, suggesting that *O*-GlcNAcylation plays a role in the essential recognition events in nuclear transport [13].

### 2.6. Chemoselective Reactions Involving Diazirine-Functionality

**Figure 5:**
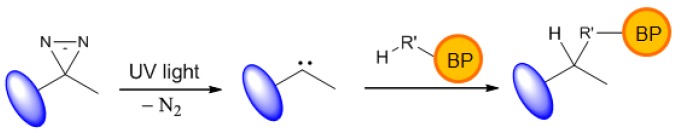
Diazirine reaction scheme for UV-induced photochemical conjugation. BP represents the binding proteins.

Photo-activatable diazirine reaction chemistry: Photo-reactive crosslinking chemistries are used widely for non-specific bioconjugation. Although various photo-reactive groups have been introduced, the two most common groups are diazirines and aryl azides. Compared to aryl azides, diazirines are a newer class of photo-activatable chemical groups and much smaller in size that can allow their incorporation into the proteins of interest in the form of a metabolic chemical reporter. Diazirines have better photostability than phenyl azides, and they are activated more efficiently with long-wave UV lights. Upon UV radiation, a reactive carbene intermediate is formed and this reactive intermediate can form covalent bonds through an addition reaction with any neighboring molecules (Figure 5).

### 2.7. Important Aspects of Consideration for Metabolic Chemical Reporters

The utility of metabolic chemical reporters has been expanded widely to the majority classes of posttranslational modifications. Ideally, metabolic chemical reporters are expected to enter a single biosynthetic pathway, resulting in the labeling of one type of modification. However, as described earlier, synthetic analogs developed for labeling *O*-GlcNAc label *N*-linked and/or *O*-linked glycans as well. For example, UDP-GlcNAz produced from unnatural GlcNAz metabolism is interconverted enzymatically to UDP-GlaNAz, resulting in incorporation into other classes of glycoproteins [14]. In addition, carbohydrate MCRs might enter the branch metabolic pathways before the formation of their corresponding UDP-sugar analogs. One discovered branching pathway involves a protein acetylation metabolism from the GlcNAc salvage pathway. An understanding of this “metabolic crosstalk” is essential to decipher the biological functions of a single type of glycosylation.

## 3. Chemical Reporters and Their Chemistries for Labeling *O*-GlcNAc In Vitro

### 3.1. Ketone-Functionalized Chemical Reporter for Labeling O-GlcNAc In Vitro

**Figure 6:**
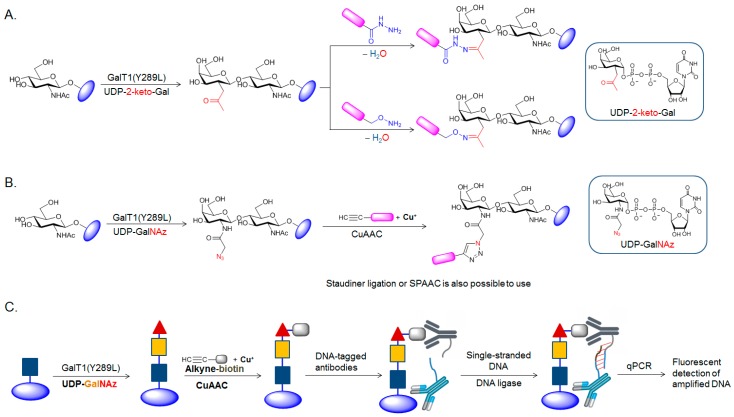
Schematic description of chemoenzymatic tagging of ketone- (**A**) or azide-functionality (**B**) followed by its bioorthogonal ligations to probe the *O*-GlcNAcylated proteins, and “Glyco-seek” strategy (**C**).

Ketone-bearing monosaccharides are the first generation of the metabolic chemical reporters for studying glycoproteins [29,55]. The unnatural monosaccharides carrying ketone functionality have been used to incorporate cell surface glycoproteins metabolically [29,55], but its metabolic *O*-GlcNAc analogs for the incorporation of *O*-GlcNAcylated proteins in cells have not been reported. In general, ketone condensation reactions have rather slow kinetics (*k*_2_ = 10^−4^ to 10^−3^ M^−1^ s^−1^) [55,56] requiring a high concentration of nucleophile reagents to obtain good labeling, which might lead to cell toxicity and background signal. In addition, carbonyl condensation takes place under weak acidic pH conditions, which is difficult to achieve inside most cellular compartments. Therefore, ketone condensation reactions are believed to be best suited for probing in vitro or cell-surface labeling rather than probing the intracellular biomolecules in cells, including protein *O*-GlcNAcylation. A few methods can introduce carbonyl functionalities to the glycoproteins. Although the chemical oxidation method does not provide the specific labeling of *O*-GlcNAc with ketone/aldehyde functionalities [57], the chemoenzymatic method allows for the specific incorporation of ketone functionality to the *O*-GlcNAcylated proteins [58,59]. In the chemoenzymatic labeling method, ketone-functionalized nucleotide sugar analog (referred as UDP-2-keto-Gal) has been used for *O*-GlcNAc proteomic studies from cell lysates (Figure 6A) [58,59]. This strategy uses an engineered mutant galactosyltransferase GalT1 (Y289L), which has the enlarged donor-substrate-binding pocket thereby enhancing the substrate tolerance of the enzyme toward the more sterically hindered sugar-donor substrate, such as the ketone isostere of GalNAc (2-keto-Gal) or GalNAz onto *O*-GlyNAcylated proteins [58,59]. UDP-2-keto-Gal has been used in a number of *O*-GlcNAc proteomic studies [58,59,60]. For example, Khidekel et al. exploited a combination method of chemoenzymatic tagging using UDP-2-keto-Gal and the mutant GalT1 and isotopic labeling strategy to identify and quantify changes in *O*-GlcNAcylation of proteins in the cultured cortical neurons from embryonic rats upon an OGA inhibitor treatment [59]. The authors discovered several proteins (e.g., translational initiation scaffolding eIF4G, OGA, transcriptional repressor p66β and zinc finger RNA-binding protein) which increased in the *O*-GlcNAcylation level in the range of 24–40-fold, suggesting that *O*-GlcNAcylation is dynamically modulated by excitatory stimulation of the brain in vivo [59].

### 3.2. Chemoselective Reactions Involving Ketone-Functionality

Carbonyl condensation with hydrazide or alkoxyamines: Carbonyl functionality in ketones and aldehydes are the first functionality to be explored as bioorthogonal reporters [55]. These react with strong α-effect nucleophiles, such as hydrazines, hydrazides, and alkoxyamines [61,62]. The α-effect refers to the enhanced nucleophilicity of an atom arising from the presence of an adjacent (α) atom with a lone pair of electrons. Ketone condensations with those nucleophiles have been shown to have second-order rate constants, ranging from 10^−4^ to 10^−3^ M^−1^ s^−1^, which are lower than the other orthogonal chemical reactions described above. On the other hand, the conjugation products of carbonyl conjugations (i.e., hydrazones, acyl hydrazones, and oximes) are sufficiently stable to be used widely in protein-labeling applications.

### 3.3. Azide-Functionalized Chemical Reporter for Labeling O-GlcNAc In Vitro

The azide-labeled UDP-GalNAc analog (UDP-GalNAz) has also been introduced in the chemoenzymatic labeling substrate (Figure 6B) [63] and it has become a popular method for imparting azide-functionality to *O*-GlcNAcylated proteins in vitro in a larger number of *O*-GlcNAc proteomic studies [63,64,65,66,67]. All three azide-involving chemistries (Studinger ligation, CuAAC, and SPAAC) can be employed after chemoenzymatically installing the GalNAz moiety to the *O*-GlcNAcylated proteins, even though only CuAAC reactions have been reported in experiments involving chemoenzymatic strategy [65,66,67]. By exploiting the chemoenzymatic approach using UDP-GalNAz and mutant GalT1 combined with CuAAC reaction, Hsieh-Wilson et al. discovered that phosphofructokinase 1 (PFK1), a major regulatory enzyme that controls glucose flux through glycolysis [68], was *O*-GlcNAcylated at the S529 site under tumor growth conditions [69]. In their study, hyper *O*-GlcNAcylation of PFK1 inhibited its enzymatic activity and redirected the metabolic flux through the pentose phosphate pathway to produce the metabolites essential for DNA biosynthesis and antioxidants to quench the reactive oxygen species, which are critical for cancer cell proliferation and survival [69].

Recently, GalNAz incorporation using the chemoselective labeling method has been combined with a DNA polymerization technique to increase the detection sensitivity of *O*-GlcNAc to the attomole levels [70]. A novel strategy called “Glyco-seek” sequentially employs chemoenzymatic labeling of the chemical reporter, affinity probe bioorthogonal conjugation, proximity ligation, and quantitative polymerase chain reaction (PCR) [70]. In an example of *O*-GlcNAc detection (Figure 6C), first the *O*-GlcNAcylated proteins are labeled with GalNAz using the chemoselective method and then reacted with a biotin-alkyne probe through CuAAC to conjugate the biotin epitope. The use of two independent DNA-tagged antibodies specific for biotin and the target protein allows the attached single-stranded DNA into proximity if the target protein is *O*-GlcNAcylated. Following DNA-ligation and quantitative PCR, the protein’s *O*-GlcNAcylation can be measured. The ultrahigh detection sensitivity of this technique allows dynamic *O*-GlcNAcylation in low abundance proteins, which have not been detected. For example, Bertozzi et al. measured both the *O*-GlcNAc level on endogenous c-Rel protein, a member of the nuclear factor κB transcription factor family, and the protein expression level after a treatment of Jurkat cells with the specific OGA inhibitor Thiamet G (TMG) [70]. C-Rel was shown to be modified with *O*-GlcNAc at the S350 site, which is required for its DNA binding and transactivation functions [71]. The authors discovered that the TMG treatment increased *O*-GlcNAcylation on c-Rel significantly, whereas the c-Rel protein level remained unperturbed [70]. One limitation of this technique is that it may be unsuitable for discovering new *O*-GlcNAcylated proteins.

## 4. Conclusions and Future Perspective

Since the first discovery of protein *O*-GlcNAcylation in 1984 [72], remarkable advances have been made in identifying which proteins are modified by *O*-GlcNAc and which pathways are affected by this modification. Novel bioorthogonal chemical reporters developed for the incorporation of *O*-GlcNAcylated proteins and the new conjugation chemistries with the epitope probes highlighted in this review have been providing powerful tools to track these biomolecules in living systems and illuminating unknown aspects of *O*-GlcNAc biology. Although recently reported MCRs have higher specificity for *O*-GlcNAcylation labeling and detection, they do not escape from “off-target” labeling completely. Therefore, continued improvements in the incorporation efficiency and specificity of MCRs to achieve exclusive labeling of *O*-GlcNAc will be needed to advance their application in *O*-GlcNAc study. In addition, Glc-6-Phosphate, an intermediate in the hexosamine biosynthetic pathway enters another metabolic pathway involving protein acetylation [73]. Therefore, another area of future research will be to investigate the possible metabolic pathways of each known MCR to avoid false interpretations. The novel and even more efficient bioorthogonal conjugation reactions between chemical functionalities and epitope-conjugated probes could also be developed. Continued progress toward more sensitive and specific strategies, which are accessible, biocompatible, and simple to use, will help to discover the unknown *O*-GlcNAcylated proteins in low abundance that have been elusive using previous detection techniques. For example, a recently developed “Glyco-seek” strategy allows researchers to detect protein *O*-GlcNAcylation down to unprecedented attomole levels. Further developments and applications of chemical reporters and bioorthogonal labeling methods should help translate the information encoded in the *O*-GlcNAc proteomes into their biological function. One function of protein *O*-GlcNAcylation is its ability to regulate various protein–protein interactions, for which photoactivatable GlcNAc analogs are anticipated to provide considerable insights.

Although the past decade has seen considerable progress in the understanding of *O*-GlcNAc biology, many questions remain unanswered or partially acknowledged. Answers on “how” *O*-GlcNAc enzymes (OGT and OGA) recognize their numerous substrates; how *O*-GlcNAc modulate protein–protein interactions in time as a response to cellular stimuli, thereby affecting the diverse proteins’ functions in the cell; and how cells maintain *O*-GlcNAc homeostasis are still far from complete. In addition, the role of *O*-GlcNAcylation in Alzheimer’s disease and other neurological disorders as well as its role in transcriptional and epigenetic regulation are not completely understood. To answer these questions, many goals include improved OGT and OGA specific inhibitors in cells, improved MS detection and data-analysis tools, and site-specific *O*-GlcNAc antibodies. Cross-disciplinary collaborations between chemists and biologists should be effective in advancing this thriving field and uncovering the key mechanisms of human diseases for therapeutic intervention.
